# Viral Proteins Acquired from a Host Converge to Simplified Domain Architectures

**DOI:** 10.1371/journal.pcbi.1002364

**Published:** 2012-02-02

**Authors:** Nadav Rappoport, Michal Linial

**Affiliations:** 1 School of Computer Science and Engineering, Hebrew University of Jerusalem, Jerusalem, Israel; 2 Department of Biological Chemistry, Institute of Life Sciences, Hebrew University of Jerusalem, Jerusalem, Israel; 3 The Sudarsky Center for Computational Biology, Hebrew University of Jerusalem, Jerusalem, Israel; TUM, Germany

## Abstract

The infection cycle of viruses creates many opportunities for the exchange of genetic material with the host. Many viruses integrate their sequences into the genome of their host for replication. These processes may lead to the virus acquisition of host sequences. Such sequences are prone to accumulation of mutations and deletions. However, in rare instances, sequences acquired from a host become beneficial for the virus. We searched for unexpected sequence similarity among the 900,000 viral proteins and all proteins from cellular organisms. Here, we focus on viruses that infect metazoa. The high-conservation analysis yielded 187 instances of highly similar viral-host sequences. Only a small number of them represent viruses that hijacked host sequences. The low-conservation sequence analysis utilizes the Pfam family collection. About 5% of the 12,000 statistical models archived in Pfam are composed of viral-metazoan proteins. In about half of Pfam families, we provide indirect support for the directionality from the host to the virus. The other families are either wrongly annotated or reflect an extensive sequence exchange between the viruses and their hosts. In about 75% of cross-taxa Pfam families, the viral proteins are significantly shorter than their metazoan counterparts. The tendency for shorter viral proteins relative to their related host proteins accounts for the acquisition of only a fragment of the host gene, the elimination of an internal domain and shortening of the linkers between domains. We conclude that, along viral evolution, the host-originated sequences accommodate simplified domain compositions. We postulate that the trimmed proteins act by interfering with the fundamental function of the host including intracellular signaling, post-translational modification, protein-protein interaction networks and cellular trafficking. We compiled a collection of hijacked protein sequences. These sequences are attractive targets for manipulation of viral infection.

## Author Summary

Many studies focused on the exchange of genetic material between viruses and cellular hosts. The diversity of viruses argues that, along the evolutionary history, viruses have shaped the host genomes. While most viruses have many opportunities to exchange genetic material with their hosts, tracing such events is challenging as the origin of the sequences is masked by the high mutation rate of many viruses. On the other end, for completing a successful infection cycle the viruses must cope with the cell machinery for entry, replication and translation while hiding from the host immune system. We collected evidence for instances of viral protein sequences that were most probably “stolen” from the hosts. Additionally, a shared ancestry with metazoa is associated with 670 Pfam domain families. For half of these families, the origin of the viral proteins from its host is supported. For about 75% of the cross virus-metazoa families, the viral proteins are significantly shorter than their counterpart host proteins. Most of these cross-taxa viral proteins are single domain proteins and proteins with a simple domain composition relative to the proteins of their hosts. These viral proteins provide insights on the overlooked intimacy of viruses and their multicellular hosts.

## Introduction

Many studies, mainly from bacteria and unicellular eukaryotes, focus on the exchange of genetic material between viruses and cellular hosts. Sequences are best studied through their structural and functional domains [1], [2], [3], [4], [5]. The evolution of domains is a significant force for shaping the proteins along the tree of life. Sequence exchange between genomes within and between superkingdoms is evident from the appearance of a domain in a particular phylogenetic branch [6]. The contribution of horizontal gene transfer is not limited to bacteria but has occurred across distant species [3]. For example, some signaling domains in bacteria are the consequence of a horizontal gene transfer [7].

The viruses are parasitic agents that maintain an intimacy with their host cells. Consequently, an extensive horizontal evolution [8] is associated with the viral life cycle. The lack of similarity of viral proteins (e.g., capsid proteins) with any cellular organisms is in accord with their early and unique origin [8], [9]. Most likely, the modern viruses originated at the early RNA world of the primordial genetic pool.

With the increasing numbers of sequenced viruses, similarity among seemingly unrelated viruses was reported. A role of the hosts as vehicles for such cases is proposed. For example, the structural similarities observed between bacterial viruses (PRD1, Bam35), Chlorella virus (PBCV-1) and adenovirus in the coat proteins, led to the proposal that all viruses are old, probably preceding the cellular life. Furthermore, it is compatible with polyphyletic virus origins, as opposed to the monophyletic origin of cellular life [10]. Still, assignment of viruses to the phylogenetic tree of life remains unresolved [11].

Notably, viruses as vectors (mainly RNA viruses) have the potential to rearrange the genomic material, and thus, to change the domain architecture [12], [13], [14]. Studies on horizontal gene transfer focused primarily on viruses infecting bacteria and archaea (e.g., bacteriophages) [15], [16]. The co-evolution of viruses toward their hosts indicates an active crosstalk on an evolutionary time scale [17], [18], [19].

Several studies reported on a handful of cases of functional mimicry by viral proteins [20]. In few cases, evidence for gene transfer from the host to the virus is obvious. For example, the photosynthetic efficiency in cyanobacteria (Synechococcus and Prochlorococcus) relies on components of the photosystem II. These critical components express in the respective phages [21]. In the case of the phytoplankton–virus system, the DNA virus EhV that infects the microalge (Emiliania huxleyi), contains a complete metabolic pathway as a result of a horizontal gene transfer [22]. A similar case is demonstrated for the dUTPase genes (Dut) that are necessary for regulating the cellular levels of dUTP. Phylogenetic analysis revealed the origin of the viral Dut sequence in a monophyletic cluster of DNA viruses with eukaryotic hosts [23].

The Acanthamoeba polyphaga Mimivirus and the family Phycodnaviridae [24], contain many genes that are found in cellular organisms. For example, the giant virus Cafeteria roenbergensis virus (CroV) includes numerous eukaryotic-like genes for translation factors, ubiquitin pathway components, intein elements, histone acetyltransferase and more [25]. These are extremely large viruses of aqueous environments that infect bacteria, animals and protists [26].

A search for similarities between viral and host proteins has largely been focused on herpesviruses [27], Hepadnaviridae [28] and others. However, the high mutation rate of RNA viruses [29] and the coexistence between viruses and their hosts for millions of years has most likely blurred the sequence similarity. Recently, several studies challenged the origin of ancient viral segments in metazoan genomes. These sequences that are called EVE (for endogenous viral element) encompass all virus-derived genomic loci [30].

In this paper, we present a coherent survey on protein sequences that are shared between viruses and their hosts. We assess the scale of the phenomenon by focusing on the viral-related protein sequences that appear in metazoa. We have used the current archive of all proteins [31] as the basis for identifying sequences with a potentially common origin. Presumably, their appearance in the virus reflects virus-acquired sequences. Of about 190 instances of highly similar viral-eukaryotes sequences, we recognize that only a small number originated from a host origin. We extended the collection of viral proteins that have a host origin by investigating the eukaryotes-viruses Pfam families [32].

We focused on the 670 Pfam cross-taxa families that contain viruses and metazoa. A careful examination reveals that these instances reflect either missed annotations or the remnants of sequence exchange by virus infection. To distinguish these possibilities, we constructed sequence alignment trees for all 670 Pfam families. From the properties of the trees, we focused on 335 families that most likely contain viruses that hijacked sequences from their host. We found that most of the viral proteins in the orthologous families are much shorter and composed of simpler domain architectures. In almost all cases, the number of domains and the sequence of the tails and the inter-domain linkers are considerably shorter in the viral proteins relative to their counterpart host proteins. We discuss the potential of such short viral proteins to interfere with critical cellular functions and thus are candidates for manipulation strategies in defeating viral infection.

## Results/Discussion

### Genetic material exchange between viruses and their hosts

**Figure 1:**
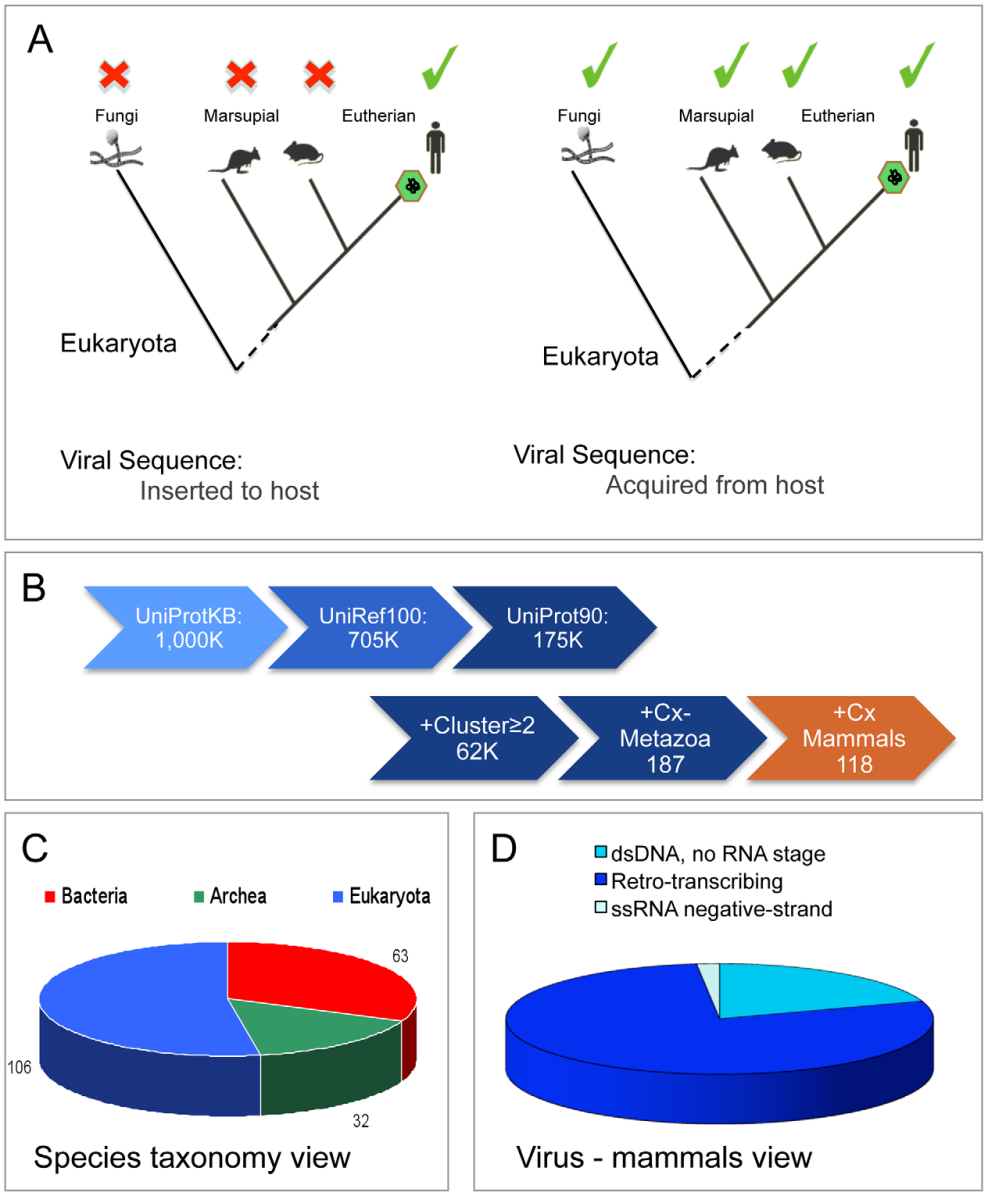
Lateral gene transfer between virus and host. (**A**) Two simplified scenarios in support of a genetic exchange event from the virus to the host genome (left) and from the host to the virus genome (right). A homologue of a viral protein in a eukaryote is marked by a green check mark and a red X symbol when no homologous sequence is detected. (**B**) The sequential filtration steps applied from UniProtKB to the set of UniRef90 viruses-mammals cross-taxa clusters. The numbers indicate the size of the dataset after filtering. (**C**) A species perspective on cross-taxa from the UniRef90 clusters that contain viruses and eukaryotic proteins. The division is according to the 3 superkingdoms. The number of the different species that are represented from each superkingdom is indicated. (**D**) The partition according to the classes of the viruses for UniRef90 clusters that contain viruses and their mammalian hosts (∼2500 proteins). The main viral families that infect vertebrates according to their replication classes are listed in Supplementary information, Table S1.

Figure 1A shows two over-simplified scenarios in support of a genetic exchange from the virus to the host genome and in the reverse direction, from the host to the viral genome. In the first scenario, a viral sequence is detected in the host (e.g., human) but not in the rest of the phylogenetic branch. The following scenario accounts for viral sequences acquired from the host (Figure 1A, right). Under this scenario, the viral gene sequence is identified in a broad group of organisms that belong to a phylogenetic tree that includes the host (human). Therefore, the sequence in the virus is most likely a reflection of a hijacking event, according to an argument of maximum parsimony.

Supporting evidence for the directionality of the genetic exchange of viral and cellular organisms relies on a detailed phylogenetic analysis. The topology of the reconstructed tree is used to support the most parsimonious scenario (see Materials and Methods). The simplified illustrations in Figure 1A do not address the more complicated, realistic instances in which different viruses carry sequences that resemble various organisms. An additional criterion used in supporting the occurrence of sequence acquisition by viruses is the presence of a sequence resemblance in the known host. The origin of viruses is probably preceding the cellular life [8], [10]. Thus, the ancient events in which viral sequences were incorporated into an ancestor eukaryote cannot be traced by their sequence similarity. Still, a conserved functional or structural similarity could expose such early events [33]. In this study, we have not attempted to date the horizontal transfer event. Furthermore, we will not discuss the events of genetic material exchange (see discussion in [34]), but limit our study to the acquisition of coding sequences in viruses and metazoa.

There are about one million viral proteins in the UniProt database (990,049, August 2010) that represent about 66,000 viral strains. This is a highly redundant resource and about half of it composed of medically relevant strains including Hepatitis B viruses (HBV) and Human immunodeficiency virus (HIV). We took advantage of a reliable source of UniRef [35] that unifies sequences according to their identity level along the sequence length. We used UniRef90 classification (see Materials and Methods). There are >165,000 UniRef90 clusters that contain at least one viral protein (Figure 1B). However, from this set, we only considered 262 instances that contain at least two proteins, where one of them must be a eukaryote (Figure 1B). Of the 5,482 cross-taxa clusters that contain sequences from viruses and cellular organisms, 95% are sequences of bacteriophages and plasmids confined to the bacteria [36]. We will not further discuss the events that are confined to bacteria and archaea.

A taxonomical view shows the diversity of the organisms that share the UniRef90 clusters with viral proteins (Figure 1C). It shows that the eukaryotes are the most diverse group with 106 species that share their homologues with viral proteins. This result suggests that the phenomenon of shared sequences is quite broad, and many eukaryotes have been subjected to a genetic material exchange. Among the UniRef90 clusters that contain viruses and eukaryotes (Figure 1B), ∼70% are from tetrapoda, 13% plants, 13% arthropoda, 4% fungi and only a smaller percentage of other taxa. We focused on the cross-taxa clusters of viruses and mammals (118 clusters include ∼2,200 proteins, Figure 1D). Viruses from Class I (dsDNA viruses with no RNA stage) and class VI (Retro-transcribing ssRNA, Plus strand) are prevalent among those that infect mammals [17]. The dominating Class VI viruses are characterized by their ability to integrate sequences into the host (Figure 1A, left). Table 1 lists the Class I viral proteins that share sequences with mammals (23 clusters, Figure 1D). In Class I viruses, the virus enters the nucleus before its replication (with the exception of Poxvirus family) and its infectivity is strongly dependent on the host cell division.

**Table 1:** List of UniRef90 clusters that include mammals and dsDNA viruses (Class I).

UniRef90 Accession	Cluster name	Clus. Size	# Species	# Viral proteins	Bac origin^a^	Seed Length^b^	Support^c^	H2V^d^
Q02582	Antitermination protein	108	103	13	+	207	Cont	NO
Q7YQE1	Beta-1,3-galactosyl-O-glycosyl	12	9	7	−	440	Bovidea	YES
Q9IEZ9	E1A nucleoprotein (Frag)	6	2	4	−	12	Cont	NO
Q4VHD0	Interleukin 10 (Frag)	10	5	5	−	68	Mammal	YES
Q6PVS0	Interleukin 10 (Frag)	2	2	1	−	45	Caprinae	YES
P43480	Interleukin-10	13	10	2	−	178	Pecora	YES
Q64142	T1 antigen (Frag)	41	2	39	−	134	Cont	NO
O75978	L1 capsid protein (Frag)	5	4	4	−	100	Cont	NO
O75979	L1 capsid protein (Frag)	2	2	1	−	105	Cont	NO
P03073	Large T antigen	4	4	3	−	785	Cont	NO
P36741	Major capsid protein L1	9	2	1	−	539	Cont	NO
P03076	Middle T antigen	4	4	3	−	421	Cont	NO
B9EHT2	Olfr780 protein (transposase)	119	82	2	+	402	N.D.	N.D.
P06426	Probable protein E5	3	2	1	−	75	Cont	NO
P06463	Protein E6	36	2	1	−	158	Cont	NO
P21735	Protein E6	8	2	1	−	158	Cont	NO
P06788	Protein E7	10	2	1	−	105	Cont	NO
P21736	Protein E7	6	2	1	−	106	Cont	NO
P06956	Recombinase cre	28	14	18	+	343	Cont	NO
Q6QMZ0	Ribosomal S27a (Frag)	33	28	1	−	115	N.D.	N.D
P68834	Small T antigen	10	9	8	−	195	Cont	NO
Q8SWD4	Ubiquitin	217	158	119	−	77	Euk	YES
P0C6Z6	Viral IL-10 homolog	4	2	1	−	170	Mam	YES

a Bac, Cluster is mixed with bacterial proteins.

b Length of cluster's seed protein.

c Analysis is based on phylogenetic tree and analyzing the expanded cluster according to UniRef50.

d H2V, from host to virus. I.e., sequences acquired by the virus from a metazoan host. N.D. Unresolved; Cont, contamination; Frag, Fragment.

Discriminating whether a sequence has originated from the virus or the host is not straightforward. We generated for each cluster a phylogenetic dendogram and analyzed the connectivity of the viral protein in view of its neighboring sequences. Often the analyzed cluster is too small. In such cases, we expanded the cluster to the relaxed UniRef50 classification. We applied additional criteria in support of a virus having acquired protein sequences from the host: (i) The tested sequence appears in several organisms (≥2) on the same evolutionary branch (as in Figure 1A, right); (ii) The tested sequence is not associated with viral contamination. Most analyzed cross-taxa clusters derive from the contamination by viral proteins following integration of the virus to a mammalian genome. Several instances were contaminated by the extensive use of viral vectors as vehicles in variety of molecular manipulations (e.g., Adenoviruses, Table 1).

Another source of contamination is from cancerous cells infected by viruses (e.g., human papilloma virus). In such instances, some sequences that are assigned as ‘human’ are incorrectly annotated. In these instances that reflect the incorporation of the virus to the host, different protein sequences from the same virus are identified which are best explained as a result of infection or an integration event. For example, the proteins in UniRef90 clusters P06426, P06463, P21735, P06788, P36741 and P21736 belong to Human papilloma virus (Table 1).

Studies on the viral sequences that were integrated into the vertebrate germ line and hence shaped the vertebrate genetic heritage were reported [37], [38], [39]. Herein, we only consider the protein sequences that are shared by viruses and their metazoan hosts. The principal virus families that infect multicellular eukaryotes are listed in Supportive data Table S1.

### A small set of highly conserved genes acquired by viruses

**Figure 2:**
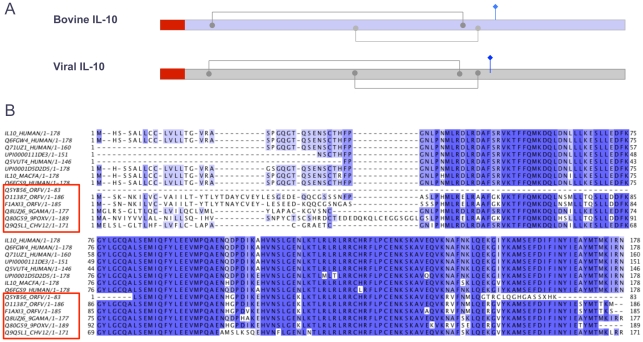
Multiple sequence alignment for Interlukin-10 (IL-10). (**A**) Functional features of human IL-10 include the disulfide bridges, glycosylated modified residues (blue, diamond line) and the signal peptide (red frame). Most viral proteins in the cluster share the 180 amino acid proteins. (**B**) Multiple sequence alignments (MSA) from viral representatives (red frame) and various mammals are shown. The viral proteins include Orf virus (ORFV), Macacine herpesvirus 4 (Rhesus lymphocryptovirus), Bovine papular stomatitis virus and Cercopithecine herpesvirus 12 (CeHV-12) (Baboon herpesvirus). Weak sequence similarity is confined to the N-terminal that covers the signal peptide.

For few instances, a support exists for viruses that hijacked sequences from the host. Among Class I viruses (Table 1) the shared functions include interlukin-10 (IL-10) (Figure 2), beta-1,6-N-acetylglucosaminyltransferase (β1,6GnT) (Figure S1) and Ubiquitin. The β1,6GnT and IL-10 are found exclusively in metazoa and the multicellular eukaryotic branch. The key features and the functional amino acids are conserved in the viral and the corresponding mammalian proteins (Figure 2A, Figure S1). Indeed, in human cells lacking β1,6GnT gene, the Bovine herpesvirus 4 (BoHV-4) sequence fully recovered the missing enzymatic activity [40]. Resolving the evolution of the Ubiquitin in the genome of Pestivirus suggested that the virus hijacked Ubiquitin-related sequences in two consecutive events [41].

A browsable table is available at www.protonet.cs.huji.ac.il/virost/tables/UniRef90-Class1.html.

Figure 2 shows a prototypic case of viral proteins that resemble the host protein. Interleukin 10 (IL-10) inhibits the induction of pro-inflammatory cytokines. IL-10 was found in many viruses including Epstein–Barr virus (EBV), equine herpesvirus (EHV) and cytomegalovirus (CMV) [42]. Presumably, the gene product protects the infected cells from the host defense mechanism. An extended cluster of IL-10 (Table 1) covers 20 viruses and 96 cellular organisms (UniRef50_P22301). Representatives of viral and metazoan proteins are shown by the multiple sequence alignment (MSA) (Figure 2B). Most of the variations in the viral and metazoan protein reside in the sequence of the N-terminal that covers the signal peptide (Figure 2B). Traces of a genomic organization of the host in the viral genome were reported. For example, IL-10 like sequence from the gammaherpesvirus ovine herpesvirus 2 includes 5 exons and 4 introns [43].

Inspecting the UniRef90 clusters that contain proteins from viruses and metazoa (187 clusters) shows a wide variation in the distribution of protein lengths (Supportive data Table S2). Viruses tend to reduce their production load by deleting and reducing the unessential genetic material [44]. While this length reduction is an absolute necessity for most viruses, some giant viruses (e.g., Mimivirus, Chlorovirus, and Cafeteria roenbergensis virus) include ∼1000 proteins [24], [25]. The evolution origin of proteins from the Giant viruses remains unknown [45]. Still, 12% of the Acanthamoeba polyphaga mimivirus (APMV) proteins constitute a large number of host related sequences. The average length of this subset of the Mimivirus proteins (523 amino acids) is similar to the length of their homologous sequences.

### Pfam families are a rich source for tracing viral acquired sequences

The small numbers of cases of viral acquired sequences (Table 1, Supportive data Table S2) may indicate the sequence divergence that had occurred throughout evolution. We therefore expanded the analysis for remote homologous. We questioned whether the viral protein sequences that were already substantially diverged due to a rapid evolution rate, or a long evolutionary history still maintain the host protein's functional domain.

The Pfam provides a comprehensive resource of functional and structural families and domains. Each Pfam entry represents a statistical model with an average sequence identity of 30–40% among the members of the family. Currently, Pfam covers 11,912 families, where 1,165 families include at least a viral protein and a eukaryotic protein representative. Some Pfam families are extremely large. Among families that contain metazoa and viral proteins are ‘Helix-loop-helix DNA-binding domain’ (∼6000 proteins) and ‘Sugar transporter’ (∼12,000 proteins). Contamination of viral proteins in metazoan proteomes (e.g., Capsid, Env, Tat) occurs mainly as a result of viral vector manipulations in cell lines, leading to incorrect assignment as a viral-eukaryotic cross-taxa family. An example is the GFP family (PF01353) that we have manually removed from the analysis. To reduce such sporadic instances, we considered Pfam families having at least two metazoan proteins, resulting in a list of 667 Pfam families.

Supportive data Table S3 lists the species, composition of the domains and the proteins' length.

The relatively small numbers of cases of viral acquired sequences (Table 1, Supportive data Table S2) may indicate the sequence divergence that had occurred throughout evolution. Therefore, we expanded the analysis for remote homologous. We questioned whether the viral protein sequences that were already substantially diverged due to a rapid evolution rate, or a long evolutionary history still maintain the host protein's functional domain.

Over 300 cross-taxa Pfam families (virus-metazoa) are best explained by a viral acquisition of host sequences. Instances of lateral gene transfer between bacteria and their bacteriophages dominate many of the cross-taxa Pfam families. Other families contain genuine viral proteins contaminated by metazoan proteins.

**Figure 3:**
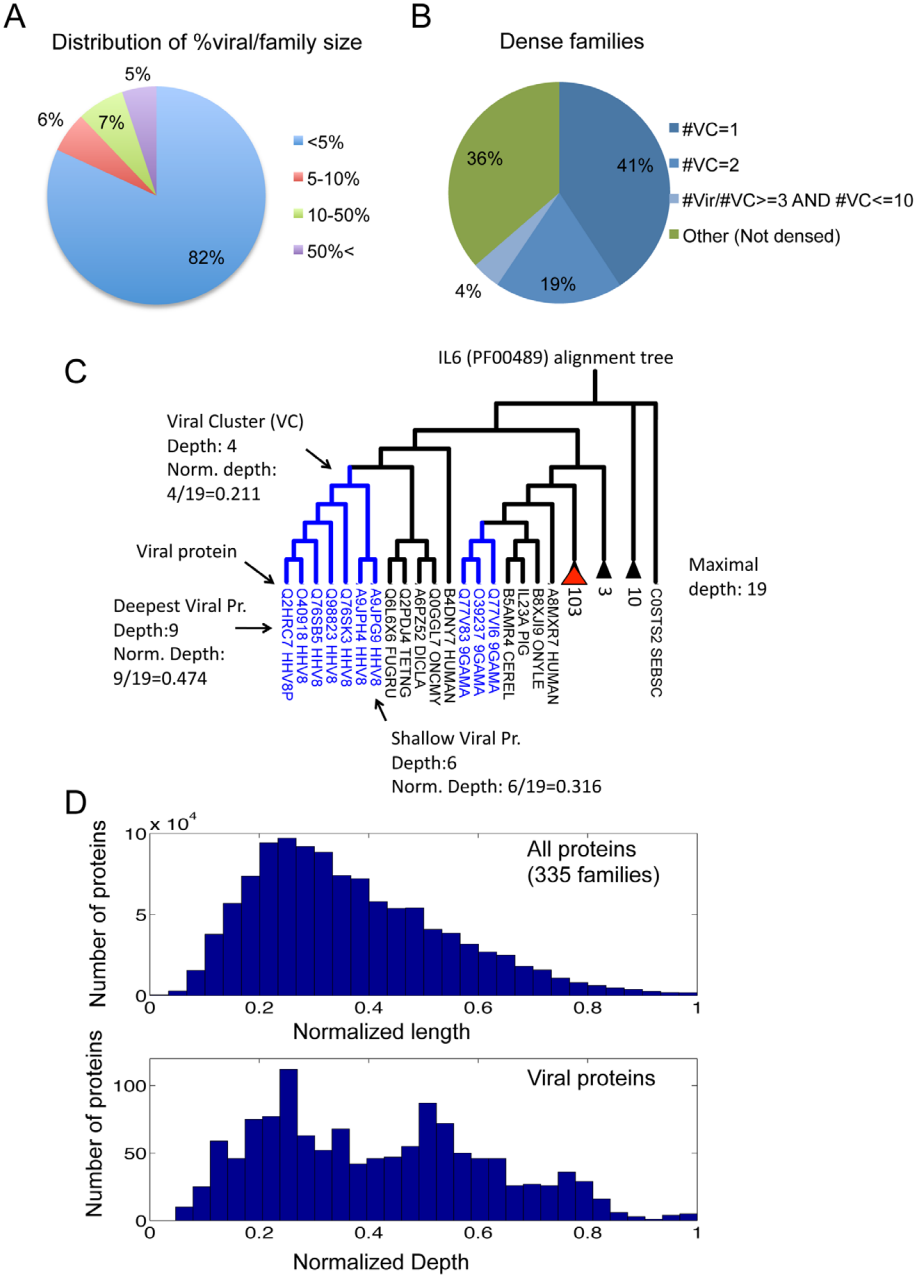
Analysis of the cross-taxa Pfam domains. (**A**) Partition of the 667 Pfam families according to the ratio of the viral proteins to all the proteins that belong to the family (in %). The vast majority (82%) of the Pfam families contain ≤5% of viral proteins (blue). (**B**) Partition of the Pfam families in which the viral proteins are clustered. VC, a cluster that includes only viral proteins in a sub-tree; #VC, the number of Viral Clusters. The analysis covers the Pfam families that contain ≤5% of viral proteins (blue slice from A). We consider only 335 Pfam families that contain the families with (i) only one VC (41%), (ii) 19% with exactly 2 VCs and (iii) 4% with ≤10 VCs but with a condensation factor ≥3 (#Vir/#VC). Using this filtration additional 36% of the families were removed (marked, others). (**C**) Alignment tree of IL-6 Pfam family (PF00489). The family contains 10 viral proteins (blue font), 2 viral clusters (#VC, blue font sub-trees) and 136 metazoan proteins. Collapsed sub-trees are represented by a triangle indicating the number of proteins as the tree leafs. The maximal leaves in this tree are 19 (in the collapsed sub-tree, red triangle). The deepest viral protein in the tree is of depth 9, and thus the normalized (norm.) depth is 9/19 = 0.474. The depth of the viral cluster (VC, the maximal sub-tree which contains only viral proteins) is 4/19 = 0.211. (**D**) The distribution of normalized depths of all proteins for the 335 Pfam families (top) and the distribution of the depths of the viral proteins in these families (bottom).

In order to justify the directionality of sequences from the hosts to the virus, we constructed for each of the 667 Pfam families a sequence-based tree (MSA based on the domain and not the full length sequence). We considered Pfam families in which only 1–2 viral proteins are included in the family, and families in which the percentage of the virus proteins in the family is small (<5%, Figure 3A). The vast majority (547 families, 82%) of the analyzed Pfam families fulfilled these criteria (Figure 3A, blue). We also requested that the viral proteins are clustered in sub-trees within the family tree. We counted the number of viral proteins spreading within the sequence alignment tree. We suggest that viral proteins that are clustered in a defined sub-tree (called Viral Cluster, VC) are likely to represent a single episode of acquired sequence from the host. Consequently, only a limited diversity among the closely related viruses is expected in view of the rest of the tree. 64% of the 547 Pfam families from the previous selection fulfill the requirement for clustered viral proteins. These are the Pfam families that contain ≤2 viral clusters (60%), and other families (4%) that are specified by a high degree of condensation (i.e. the ratio of the viral proteins to the number of VCs is ≥3). These filtration steps further reduced the list of relevant Pfam families to 335 (Figure 3B).

We show a tree constructed for one of the 335 families. The IL-6 (PF00489) family contains 10 viral proteins (Figure 3C, blue) that are split to two sub-trees of viral clusters (VC) and other 136 Metazoan proteins (marked as collapsed sub-trees). The maximal depth in this tree is 19 (included in the collapsed sub-tree, red triangle). The deepest viral protein in the tree is of depth = 9, and its normalized depth is 9/19 = 0.474. The depth of the viral cluster (VC, the maximal sub-tree which contains all viral proteins) is therefore, 4/19 = 0.211.

The normalized depth for all the proteins in the 335 Pfam families (Figure 3D, top) is analyzed in view of the distribution of the normalized depth of the viral proteins within these families (Figure 3D, bottom). It seems that the two distributions are remarkably different (Figure 3D) which is in accord with the notion that the viral proteins are relatively isolated subsets among the proteins from the cellular organisms in the relevant Pfam families.

Table 2 shows a sample of these families along with the cellular process and the protein function in the viral life cycle. A full list of the 667 Pfam families with the analyzed properties of their alignment trees is provided in Supportive data Table S3.

**Table 2:** A sample of the cross-taxa Pfam families of viruses and metazoa.

Pfam ID	Common phyla	Cluster Name	Virus family^a^	Function	# Species (Sequences)	# Viruses
PF01027	Euk-Bac	UPF0005	I-Her, I-Pox	Inhibit apoptosis	735 (1845)	12
PF02758	Chordata	PAAD_DAPIN	I-Pox	Inhibit apoptosis	34 (182)	5
PF00020	Metazoa	TNFR/NGFR	I-Her, I-Iri, I-Pox	Inflammation, apoptosis, autoimmunity	97 (853)	37
PF01403	Metazoa	Sema	I-Her, I-Iri, I-Pox	Induced B and T cell proliferation	104 (693)	28
PF00341	Metazoa	PDGF	I-Iri, I-Pox, VI-Ret	Mitogen	87 (270)	10
PF00235	Euk	Profilin	I-Pox	Interrupt actin	231 (628)	20
PF00413	Euk	Rad9	*I-Phy*	Cell-cycle arrest Transcription	93 (164)	2
PF00084	Euk	Sushi	I-Her, I-Pox	Innate and adaptive immunity	163 (6244)	43
PF07988	Vertebrate	Wos2	VI-Ret	Regulation cell cycle	15 (52)	2
PF00243	Chordata	NGF	I-Pox	Neuronal survival	805 (1684)	3

a Virus class is indicated by the Baltimore classification (Table S1). Virus families that do not infect vertebrates are shown in italic. For interactive Table see www.protonet.cs.huji.ac.il/virost/tables/Pfam.html. For a complete list see Supportive data Table S3.

### The PAAD/DAPIN/Pyrin family in view of the domain composition and viral evolution

**Figure 4:**
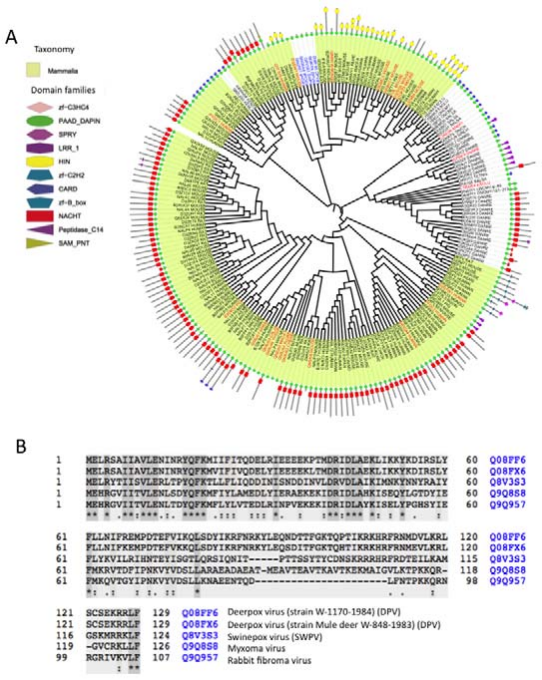
Analysis of the Pfam PAAD/DAPIN/Pyrin domain family. (**A**) PAAD_DAPIN (PF02758) is a diverse domain family at the N-terminal of all proteins. Visualization of the phylogenetic alignment tree is based on iTOL [56]. The Pfam family (182 proteins) includes 5 viruses from the dsDNA (Class I) that belong to Poxviridae. The viruses are shown in blue. Additional 19 proteins contain only the PAAD domain (red color). Each protein is shown as well as the schematic domain composition. Note that most proteins appear in the context of additional Pfam entries (158/177 occurrences). The length of proteins and the corresponding domains are indicated by their colored symbols in the outer circle. The mammalian proteins are highlighted. (**B**) MSA of the viral proteins covering the ∼80 amino acids of the PAAD domain indicating the high divergence among these viral proteins. Similarity level is shown in a gradient of gray color.

One of the families that exemplified the trend found in virus-metazoa Pfam families is the PAAD/DAPIN/Pyrin family (PAAD_DAPIN, PF02758). This domain family is a diverse family (26% average sequence identity) that includes 34 cellular species and 5 dsDNA viruses that belong to the Poxviridae. The PAAD domain is at the N-terminal regions of proteins. This domain occurs in several multicellular organisms, in the context of inflammation, signaling and apoptosis (Figure 4).

Several observations could be extracted for the PAAD domain: (i) Based on a multiple sequence alignment (MSA) of the PAAD domain sequences it is evident that the 5 viral proteins were diverged significantly (Figure 4B). All 5 viral proteins reside in one cluster, in the phylogenetic tree, together with other mammals as their sibling in the tree (Figure 4A, blue font). The domain architecture within the protein of the family is best explained by an initial extensive duplication of the PAAD domain (Figure 4A, green symbol). At present we identified ∼50 such proteins in human and mouse (Figure 4A); (ii) Most members of the PAAD family contain additional domains (in 158/177 occurrences). For example, the combination of PAAD and NACHT domains (Figure 4A, red symbol) are in 93 proteins, and PAAD, NACHT and LRR are in 2 proteins; (iii) The majority of the other domains (e.g., HIN-200, CARD) function in the regulation of apoptosis; (iv) All 5 viral proteins are single-domain proteins with PAAD domain. There are other 19 cases of the single domain proteins (Figure 4A, red font). Note that these proteins spread throughout the sequence-based tree. Presumably, it is a reflection of a domain loss event. Some of these proteins are fragments (e.g., Q5T3V8_HUMAN), and others include less characterized PfamB domains [32] (e.g., IFI4L_MOUSE, Q3UPZ5_MOUSE).

### Viral proteins that originated from the host sequences are mostly single domain proteins

The initial tests on UniRef90 covered 14,000 proteins in relatively small clusters (<90 proteins on average, Supportive data Table S2). In contrast, the collection of the cross-taxa Pfam families (Table S3) covers 161,000 viral proteins and 400,000 metazoan proteins. Therefore, focusing on the cross-taxa Pfam families provides an opportunity to increase the statistical power of the tests.

**Figure 5:**
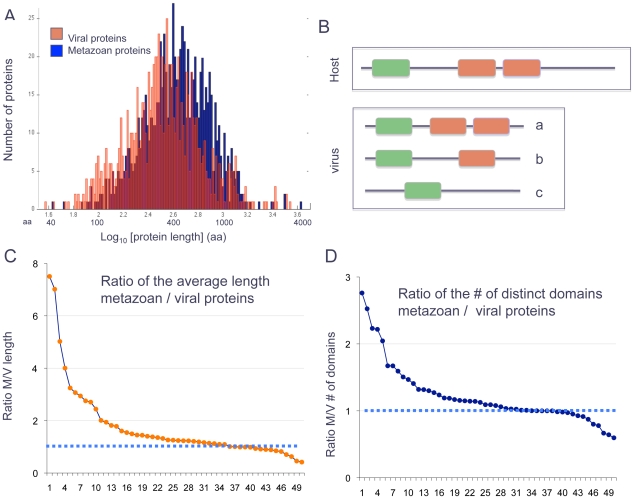
Length of proteins and Pfam domain properties of viral-metazoan proteins. (**A**) Analysis of the protein length distribution of viral (red) and metazoan proteins (blue) for the 667 Pfam based families. (**B**) A schematic view of multicellular proteins and possible variations. Changes in the viral protein length relative to the host related proteins in the same protein Pfam family could be due to a shortening of IDOL (inter-domain linkers) and the tail linkers (TAILs) (a); Additional scenarios are the elimination of a domain that is characterized by several appearances (b), and the elimination of multiple domains (c). (**C, D**) The results for 50 cross-taxa Pfam families that fulfill the following criteria: (i) at least 2 metazoan infected viral proteins, (ii) Pfam appearance is at least 3. The data points represent the proportion of the metazoan proteins' length relative to the viral proteins' length in each of the analyzed Pfam family (**C**), and the ratio of the number of the distinct Pfam domains in metazoan relative to the viral proteins (**D**). The horizontal broken line (reference line) in C and D marks no difference in the properties measured between metazoan and viral proteins (ratio of 1.0). M/V indicates metazoan/virus ratio.

Several statistical observations regarding the sequences among the cross-taxa families of viruses and multicellular organisms can be made: (i) The average length of the metazoan proteins is 507 amino acids, while the average length for the viral proteins in these families is only 396 amino acids (P-value of <1.0e-17 by the KS-test, Figure 5A). (ii) For 73% of all families, the viral proteins are shorter than the length of the average metazoan proteins in the family (P-value<1.0e-13 by the Hypergeometric test). (iii) In 67% of the families, the number of Pfam domain appearances (including several repeats of the same domain or different ones, Figure 5B) is smaller in the viral proteins relative to the metazoan proteins in the family (P-value<1.0e-40 by the KS test). (iv) In 62% of the families, the number of different Pfam domains is higher in the metazoan proteins relative to the viral proteins. (v) For the discussed families, the median number of Pfam domains is 1.06 while, for the metazoan proteins, this value is 1.7 (P-value<1.0e-32 by KS test).

### Elimination of sequence tails and internal domain in viral proteins

Many metazoan proteins are multi-domain (colored rectangle, Figure 5B). We tested whether the viral acquired sequences that belong to multi-domain proteins displayed a stronger tendency for a size reduction (see scheme, Figure 5B). A reduction in length of viral proteins may be a reflection of reducing the number of domains (Figure 5B, b–c), shortening the length of the linker sequences (Figure 5B, a) or even the trimming of the length of the domain itself.

Among the 667 analyzed Pfam families, in 103 of them, the metazoan proteins contain at least 3 Pfam domains. In 85% of this set (88 families), the viral proteins are shorter (Figure 5B, virus). Remarkably, the average length of these 103 metazoan proteins families is 912 amino acids relative to 503 amino acids for the viral proteins that belong to these families. Similarly, in this set of multi-domain proteins the viral proteins have an average of 2.9 domains, while the metazoan proteins have 4.6 domains on average (paired t-test, p-value of 1.0e-11). This shows that the tendency to reduce the protein length and the number of domains is stronger when the number of Pfam occurrence in the original host protein is higher.

In order to reduce the risk of misclassification, we further restricted the analysis to Pfam families of viruses-metazoa (with ≥3 Pfam domains) that contain at least 2 viral proteins (total of 50 families). The length of the viral proteins is significantly reduced. For 90% of these families (above the reference line, Figure 5C), the viral proteins are shorter than their matched metazoan proteins. In order to determine whether the reduction in length is due to a reduction in the number or the properties of the domains, we repeated the analysis for the ratio of the number of distinct domains (depicted by the different colored rectangles, Figure 5B) in the viral and their relevant metazoan sequences (Figure 5D). For 80% of the families (families above the reference line), number of different Pfam domains that are associated with viral proteins is reduced. Note that by this measure, a short viral protein (Figure 5B a–b) still has a ratio of 1.0. We show that the viral proteins are not only significantly shortened, but have also converged to a simpler domain composition.

The length of the individual domains between the viral and the metazoan host proteins is identical (Supportive data Figure S2). Recall, that this observation may be mainly due to the definition of belonging to a Pfam domain family.

The high statistical significance of these trends is consistent with a possibility that the short viral proteins have resulted from the acquisition of fragments from the host protein. Alternatively, it can be the result of a refinement of the acquired sequences during viral evolution. We separated each protein into three segments: (i) The Pfam domain(s); (ii) The tail linker (TAIL) that combines the amino acid extension towards the N- and the C-termini of the protein, beyond the boundary of the domain(s); (iii) The internal domain linker (IDOL) that comprises the sum of the amino acid spacers between domains. Clearly a single domain protein lacks IDOL.

**Figure 6:**
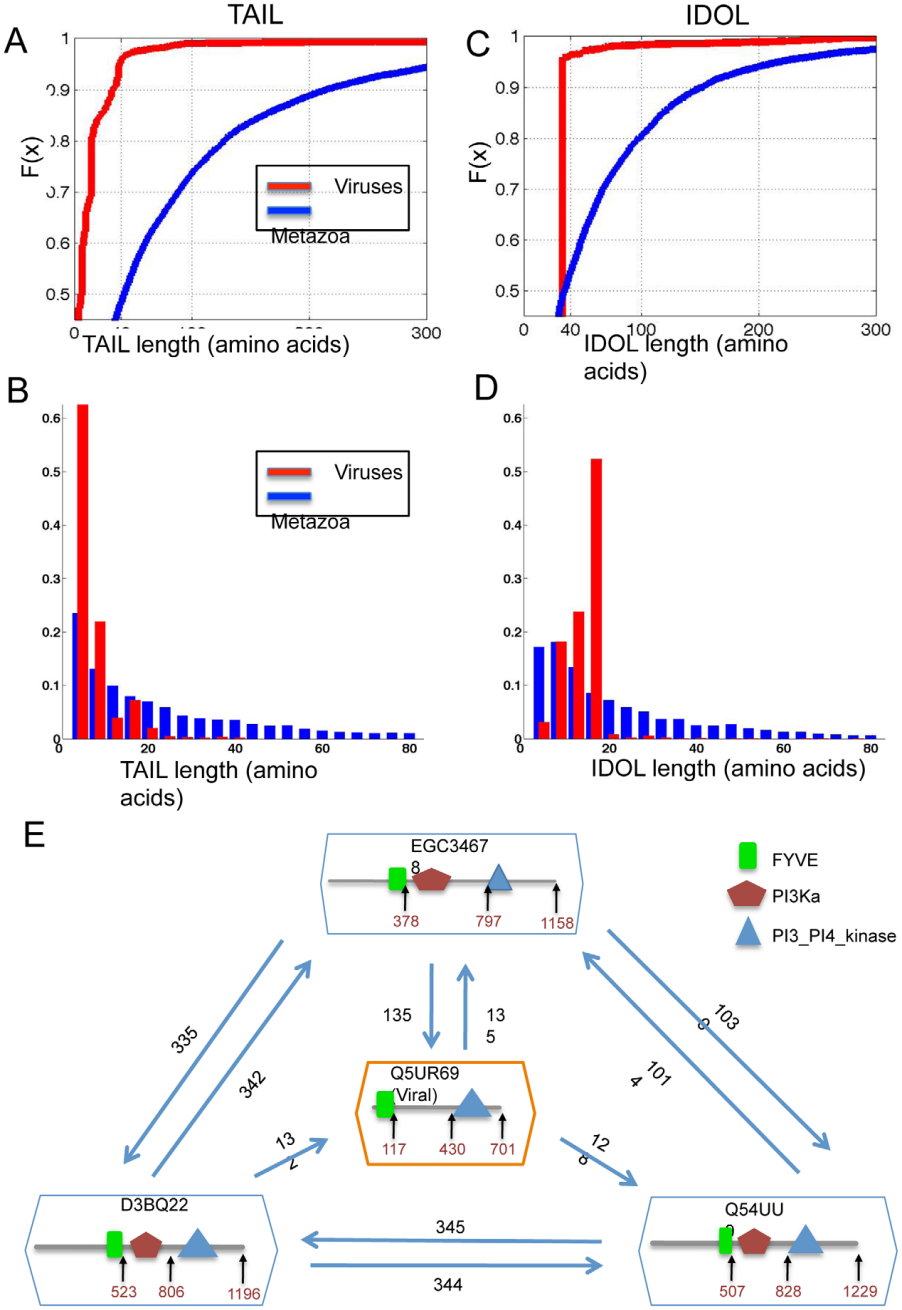
Linker and internal domain properties for cross-taxa Pfam families. (**A**) The section of the cumulative fraction function for length of <300 amino acids for the viral proteins (red) and the metazoan proteins (blue) for the tail linkers (TAILs). While 50% of the TAILs are longer than 40 amino acids in the metazoan proteins, only 3% of the TAILs are longer than this value. For the absolute cumulative fraction graphs, see Supplemental data Figure S4. (**B**) A histogram for the number of occurrences for TAIL length for viral (red) and metazoan (blue) proteins. Note that the histogram is limited to 200 amino acids. (**C**) A similar analysis as in A is shown for the inter-domain linkers (IDOLs). While 47% of IDOLs in metazoan proteins are longer than 40 amino acids, only 3% of these IDOLs are below this length among the viral proteins from the same Pfam families. For the complete cumulative graphs see Supportive data Figure S4. (**D**) A histogram for the number of occurrences for IDOL length for viral (red) and metazoan (blue) proteins. (**E**) BLAST search results for phosphatidylinositol kinase L615 (UniProt: Q5UR69) from the Acanthamoeba polyphaga Mimivirus (APMV) that infects Amoeba. The two Pfam domains: FYVE (PF01363) and PI3_PI4_kinase (PF00454) are indicated. The 3 top hit proteins are from UniProt D3BQ22, Q54UU9 and GenBank EGC34678 from Dictyosteliida. Additional of the internal Pfam PI3Ka domain is detected in the apparent host. Numbers on arrows correspond to relevant BLAST scores. The numbers in the protein frames indicate the amino acid position of the domain on the protein and the length of the protein.

We performed a separate analysis for the TAIL and the IDOL sequences (Figures 6A–6D). The study was performed on all the families that have at least 2 Pfam domains (unique or repeated). The average TAIL in viral proteins is 14 amino acids while the metazoan protein TAIL length is 85 amino acids (p-value<1.0e-150, Figures 6A–6B). Trimming of protein tails at both termini often leads to a loss of cellular localization signals (e.g., KDEL, PDZ binding sites are found at C-termini) [17]. Importantly, the average IDOL length of the viral proteins in the Pfam families is 30 amino acids, while, for the metazoan equivalent proteins, the length is 67 amino acids (p-value<1.0e-150, Figures 6C–6D). While a short TAIL may be explained by the viruses having acquired a fragmented sequence from their hosts, the same trend was found for the IDOL. Figure 6C shows that while only 54% of the metazoa IDOL have a length of <40 amino acids, in the viral proteins from the same Pfam families, 96% of the proteins have IDOL that are shorter than 40 amino acids. These results are consistent with an active trimming and refinement process throughout viral evolution. Short IDOL length is advantageous in suppressing protein misfolding, and hence, improving translation effectiveness [46].

Similarly to the finding of short IDOL sequences in viral proteins, we identified instances of an internal domain which is missing in the viral protein while the flanking domains are maintained in the same order in the eukaryotic homologous protein (Figure 6E). The viral putative phosphatidylinositol kinase L615 (UniProt: Q5UR69) is a 701 amino acid protein from the Acanthamoeba polyphaga Mimivirus (APMV) that infects Amoeba. It has two Pfam domains: FYVE (PF01363) followed by PI3_PI4_kinase (PF00454). There are no other known proteins with identical domain architecture in the Amoebozoa kingdom (there are 7 such proteins in other kingdom, e.g., Stramenopiles and Excavata). However, there are 3 proteins from the genus Dictyosteliida (slime molds) that do belong to the Amoebozoa kingdom (UniProt D3BQ22, Q54UU9 and EGC34678). In all 3 of these proteins, the architecture is composed of FYVE domain followed by PI3Ka and PI3_PI4_kinase. The missing domain of PI3Ka in the Mimivirus (APMV) provides an evidence for an active elimination of an internal domain based on parsimonious argument. The findings of shorter IDOL (Figures 6C–6D) or absence of internal domains (Figure 6E) are probably the result of the trimming and shortening of the sequences after their acquisition by the virus. The possibility of a domain insertion in eukaryotes cannot be excluded.

### Unified strategies in viral proteins acquired from the hosts

The exhaustive search for sequences that were hijacked by viruses from their host allowed us to speculate on the underlying modes of mimicry. It was shown that once a mimicry function by a virus is established, the corresponding functional partner protein of the host undergoes a fast positive selection to overcome the deleterious effect of the viral mimicry [20].

**Figure 7:**
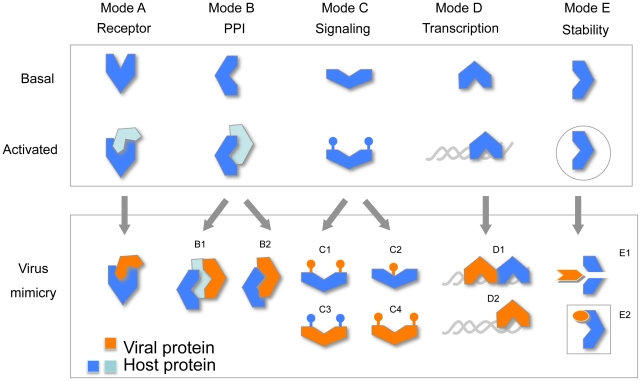
Representative strategies carried on by viral proteins acquired sequences from the hosts. The schematic modes of action (A–E) for the mimics by viral sequences are shown. The host protein (blue) and the protein-activated stage (orange) are illustrated in the top panel. Viral proteins that contain sequences that originated from the hosts may alter the host's cell through several strategies marked A1–E2. A fundamental principle is a competition with the native proteins (Modes A, B), an alteration by modifications of a target protein (Mode C), the ability to override the transcription program (Mode D) and the impact on the integrity of the target protein and the underlying cell biological processes (Mode E). The frame around the protein (in E) indicates a sub-cellular localization. See text for details.

According to these findings, the viral proteins that originated from the hosts are short versions of the full-length host proteins (Figures 5–6, Supplemental Figures S2, S4). Furthermore, these proteins are characterized by a substantial reduction in the architectures of the domains (Figure 5) and the protein linkers (Figure 6). We classified these proteins into distinct (yet not exclusive) modes of action. For simplicity, we unified the viral acquired sequences from the cross-taxa families to 5 strategy modes (Figure 7).

Mode A depicts a competition on a receptor binding by a viral ligand that replaces the natural one. Examples for this mode are the expression of the secreted IL-10 (Figure 2), IL-8 (UniProt: Q98158, Q98314, D2E2Z5) and PDGF (UniProtKB Q80GE8, Q2F842 and D0VXD7). These secreted mitogens are identified in class I and class VI viruses (Table 2).

Viral proteins participate in a rich protein-protein interaction (PPI) network [47]. Mode B illustrates PPI, where the virus uses an acquired sequence for replacing a host partner protein or for interacting with a preexisting protein complex. The result is an alteration of the cells' function. Examples for viral proteins that interfere with the host PPI are the anti-apoptotic Bcl-2 sequences and Profilin (Table 2, for example UniProt: Q5IXM3, P33828, P68695). Mammalian Semaphorins (Sema7) and the Smallpox virus A39R protein (Table 2, UniProt: Q775N9, B7SV99, Q0N658, A0ES13) share identical binding modes with a cross-reactivity towards common receptors [48].

Mode C depicts the role of protein modifications (e.g., phosphorylation). A viral protein can either mimic the host modifications (Figure 7, marked C1). Alternatively, a modification occurs by a viral enzyme (Figure 7, marked C3). Such mimicry can lead to a modification of the original site or at an entirely new site (Figure 7, marked C2). Apparently, there are instances in which both the modifying enzyme and the target proteins are both sequences that were acquired from the host (Figure 7, marked C4). This mode is dependent on the presence of active kinases (or phosphatases). For example, human cytomegalovirus (HCMV) kinase introduces phosphorylation sites that perfectly mimic the function of the cellular CDK2 (cyclin dependent kinase) [49]. An evolutionary tree alignment for viral B1R protein kinase (Supplemental Figure S3) supports the functional overlap and mimicry with the closely related cellular kinases.

Mode D depicts the importance of nucleic acid regulation of transcription. In this mode, a viral protein mimics the host regulation by either competing for an existing transcription factor (Figure 7, marked D1), or by modifying the transcription program following a DNA/RNA binding (Figure 7, marked D2). For example, the Epstein-Barr virus (EBV) encodes an activator protein that is similar to Fos/Jun family (bZIP_1, PF00170. For example, UniProt: Q80GR6, Q8QQX9, Q6USE5, D2Y5S7). The difference in specificity and the dimerization properties of the EBV activator allows the activation of an alternative transcription program [50].

Mode E collectively points to the generic strategies for damaging and deactivating the host proteins. It could be achieved by protein tagging (i.e., SUMO, ubiquitin), or the activation of viral proteases. Among the cross-taxa Pfam families, some families are associated with specialized proteases (Table S3). Mode E shows the various routes by which acquired sequences alter key cellular processes. Molecular mimicry in trafficking and the subcellular localization is common to many viruses. For example, Soluble N-ethylmaleimide sensitive factor Attachment Protein (α-SNAP) is a conserved protein among all eukaryotes. It was also found in Canarypox and Fowlpox viruses [51]. These proteins may alter the balance of the vesicular trafficking, docking and the membrane fusion machinery. In autophagy, viral proteins exploit processes such as membrane fusion and protein folding for the benefit of their replication [52].

We limit the discussion to the modes by which the shorter versions of the viral acquired proteins exhibit their impact on some cellular functions. The described modes (A–E) are effective in additional instances of molecular and functional mimicry [53], [54].

### Concluding remarks

Inspecting the viral proteome is challenging, as the majority of viral sequences are redundant and poorly annotated. Importantly, the rapid evolution and the high mutation rate in some viral classes often leads to the loss of a detectable sequence similarity and, therefore, additional cases of virus hijacking events cannot be detected based on sequence similarity search methods. Despite these drawbacks, we have traced hundreds of viral proteins with respect to their hosts. Only a small fraction of them shows high sequence similarity with corresponding host proteins. For the majority of the cases, the origin of the viral sequences and possible derivations from the host call for applying powerful models for remote homologues.

We provided analysis for 670 homologous families (according to the Pfam definition). For half of these families we provided support for sequence acquisition by the viruses from their hosts. The candidate sequences for a host to viral acquisition are useful in exploring the mechanisms by which viruses hijack and refine sequences.

We found that most of the viral proteins that potentially originated from host sequences are significantly shorter and contain fewer domains. Furthermore, we propose that the sequence refinement by the virus is a dynamic process. The inter-domain linkers (e.g., sequences connecting domains, but excluding the amino- and carboxyl tails) are significantly short, relative to other related proteins (Figure 6). The viral proteins act in the cell according to a finite number of strategies. The simpler domain composition of these viral proteins is sufficient for the utilization of functional mimicry. Currently, we are expanding the analysis by identifying short peptides in viral proteomes that serve as competition agents for neutralizing critical cellular functions.

The collections of 187 UniRef90 clusters and the 667 Pfam cross-taxa families are available as interactive tables. These tables are available at:

www.protonet.cs.huji.ac.il/virost/tables/UniRef90.html

www.protonet.cs.huji.ac.il/virost/tables/Pfam.html

## Materials and Methods

### Databases

UniProKB includes 990,049 sequences (taxonomy-viruses). The viral proteins include ∼15,000 reviewed proteins (UniProt/SwissProt). The rest of the proteins are from UniProt/TrEMBL. There are 430.6 K sequences after removal of HIV and HBV sequences. Only 241.8 K are full-length (56.1%), while the rest are denoted as ‘fragments’. The percentage of full-length proteins in metazoa is 54% (1.191 M/2.2051 M). The pre-calculated classifications of UniRef90 (i.e., identity of >90% at the amino acid level) reduce the UniProKB set to 175,236 clusters. Additional steps of filtrations are: (i) Considering only clusters with a minimal size of 2 proteins (62,129 clusters); (ii) Clusters that also include the metazoan proteins (187 clusters).

ViralZone is a database that manually assigns host-virus pairs (http://www.expasy.ch/viralzone, coordinated by UniProt/SwissProt). ViralZone holds reference strains viruses that belong to 83 families and 330 genera. This is a high quality collection of ‘complete proteome’. All viruses are classified into 7 disjoint classes (Baltimore classification index): (I) Double stranded DNA viruses; (II) Single stranded DNA viruses; (III) Double-stranded RNA and Single-stranded RNA viruses with positive and negative sense, respectively (IV, V); (VI) Positive sense single stranded RNA viruses that replicate through a DNA intermediate; (VII) Double-stranded DNA viruses that replicate via a single-stranded RNA intermediate. Major families of viruses infecting vertebrates are listed in Supporting information, Table S1.

Pfam 24.0 (11,912 families) [32] is a high quality resource for domains and families. A valid cross-taxa list was generated. Eukaryotes and viruses cross-taxa resulted in 1,165 Pfam entries. The following filtration steps were applied: (i) Pfam families with at least one viral protein and at least one metazoan protein (taxid: 33208), total of 859 Pfam families. (ii) Restricting the Pfam to families that have at least one metazoan protein and at least one metazoan-infecting virus resulted in 796 Pfam families. (iii) Pfam families with >95% viral proteins for structural element of the virus (e.g., Env, Coat, Capsid). (iv) Enzymes of the replication system were excluded, as these genes are the outcome of several events of genetic exchange [55]. Specifically, we excluded families of RNA/DNA polymerases (39 families), Exo/Endonuclease (16 families), Helicase (15 families), tRNA synthetase (8 families) and Primase (8 families). We also manually eliminated the cluster represented by the GFP (PF01353) that reflects the inevitable contamination from the extensive use of GFP as vectors in many molecular biology techniques. The filtered list includes 667 protein Pfam families (Supplemental data Table S3).

### Linker length statistics

We define linker sequences as TAILs (Tail Linkers) and IDOLs (Inter Domain Linkers). The TAILs are all sequences at the two terminals external to the first and last domain in the protein. Each protein provides two entries. The IDOL is a collection of all inter-domain sequences (excluding TAIL). Protein TAIL's length was defined as the mean of the two tail segments. In the same way, IDOL length was defined as the mean of the lengths of the inter domains linkers.

We collected the Pfam data for all proteins having at least 2 domains (i.e., having at least one IDOL) and one of the domains belong to the 667 Pfam domain families (Table S3). There are ∼57,000 such viral proteins and ∼98,000 metazoan proteins.

### Data analysis

Statistical tests were applied for the set of viral proteins in view of the host cellular protein for each cluster (or Pfam family collection). We applied statistical confidence tests (P-values) based on the non-parametric Kolmogorov-Smirnov (KS), Student t-test and the hypergeometric distribution tests. The KS test is based on the maximum distance between the two cumulative curves based on the separated viral and host proteins and viral and metazoan for the TAILs and IDOLs.

### Bioinformatics tools

Multiple sequence alignments (MSA) by ClustalW were used for constructing the Phylogenetic trees. Local alignment searches are from NCBI-BLAST. BLAST was activated with a ‘gap costs’ for Existence: 10 and for Extension: 1. The resetting of the BLAST parameters was needed for systematic identification of missing domains detection scheme. The phylogenetic trees were built using the iTol [56].

## Supporting Information

Figure S1 **Highly conserved sequences from Class I virus-mammal cross-taxa UniRef90 clusters.** (**A**) A scheme of the human β-1,6-N-acetylglucosaminyltransferase (β1,6GnT) is shown. The functional features indicated are the disulfide bridges, the glycosylation sites (diamond) and the membrane anchor domain (red box). (**B**) ClustalW based multiple sequence alignment (MSA) of β1,6GnT with representative proteins from the UniRef90 cluster UniRef90_Q7YQE1. The β1,6GnT sequences of the 2 viruses (from bovine herpesvirus type 4 (BHV-4, marked by arrows) are shown. All functional features that are shown in (A) are fully conserved.
(PPT)

Figure S2 **Statistical analysis of protein lengths and Pfam domains.** Analysis was performed for a collection of 667 analyzed cross-taxa Pfam entries (Supportive data Table S3). The graphs show the distribution of averages proteins length (two distributions per each Pfam family: one for the metazoan proteins and one for the viral proteins. A statistical KS test was performed on the domains length. No significant difference between the metazoan domains and the counterpart viral domains is detected. The same results were observed when using other statistical tests (e.g., t-test, not shown). The average and median proteins length and the average and median domain length is shown, next to the results of the statistical significant tests.
(PPT)

Figure S3 **Phylogenetic tree of the viral B1R kinase family.** A BLAST search (http://blast.ncbi.nlm.nih.gov) for the 32 highest scored proteins that belong to the B1R kinase family is shown. The query protein used is protein kinase CMLV190 from Camelpox virus. All viruses that were identified belong to dsDNA Class I of different genera. The tree branches are color coded for viruses and mammals (including platypus). All the 21 viral sequences belong to dsDNA Class I from different genera. Representatives are of Orthopoxvirus (Variola, cowpox virus) Capripoxvirus (e.g., Lumpy skin disease virus), Leporipoxvirus (Rabbit fibroma virus) and Yatapoxvirus (e.g., Yaba monkey tumor virus) and more.
(PPT)

Figure S4 **Linker lengths in Pfam families that contain viral and metazoan proteins.** The cumulative fraction function for all analyzed Pfam families for TAIL and IDOL sequences. A zoomed section of this graph is shown in Figure 6. Viral proteins are marked in red and metazoan proteins in blue.
(PPTX)

Table S1 **Baltimore Classification for the major viral families infecting vertebrate.**
(XLSX)

Table S2 **Collection and properties of the UniRef90 clusters for virus-eukaryote cross-taxa.**
(XLSX)

Table S3 **Collection and properties of the 667 Pfam families of the cross-taxa of metazoa and viruses.**
(XLSX)
