# Supplementary figures and images for: Viral Proteins Acquired from a Host Converge to Simplified Domain Architectures
Source: PLoS Comput Biol. 2012 Feb 2;8(2):e1002364. doi: 10.1371/journal.pcbi.1002364 (PMC3271019; doi:10.1371/journal.pcbi.1002364)

## Slide 1
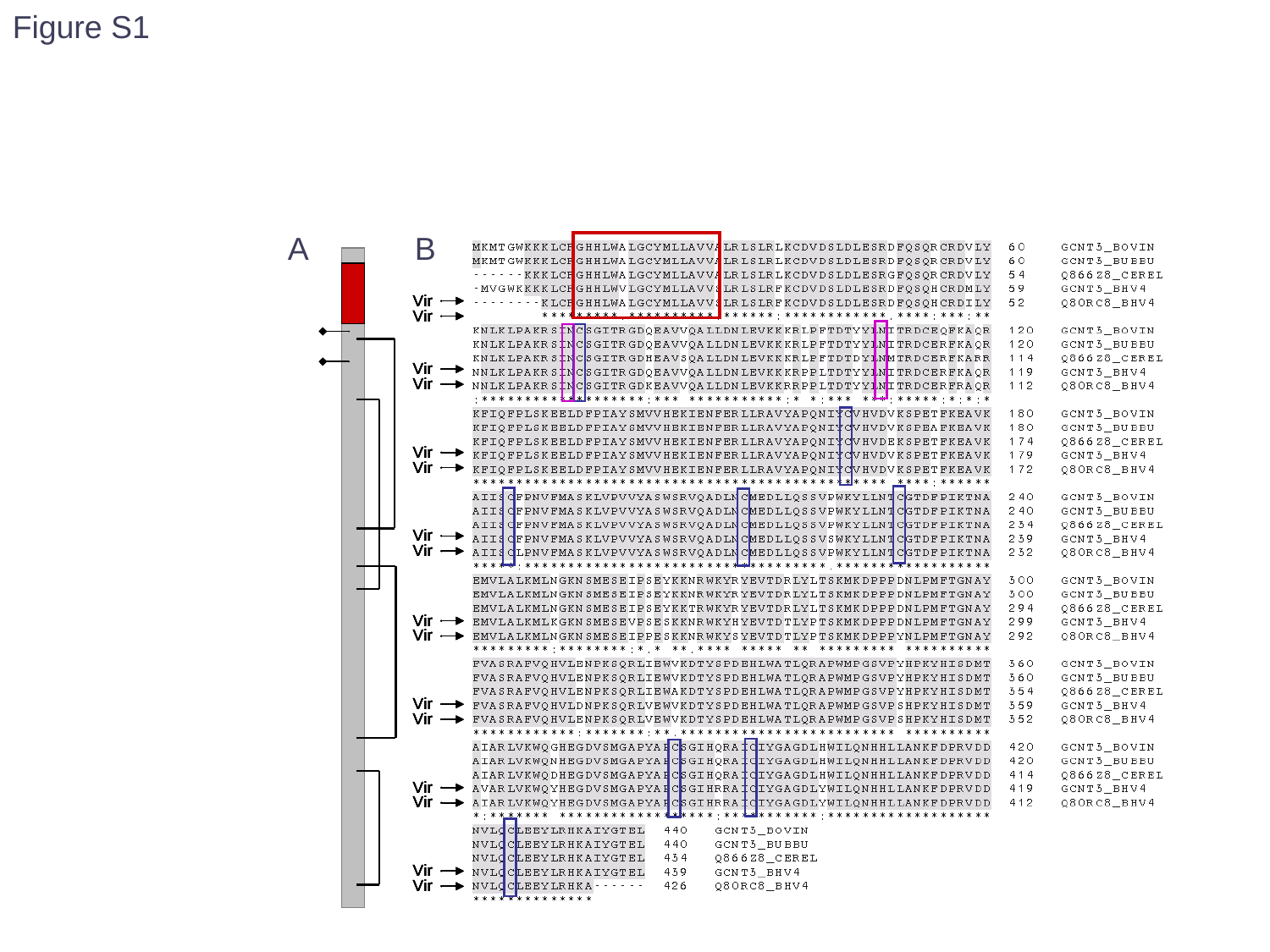

Figure S1
∂∂∂
∂∂∂
A
B

Supplement: Figure S1 — Highly conserved sequences from Class I virus-mammal cross-taxa UniRef90 clusters. (A) A scheme of the human β-1,6-N-acetylglucosaminyltransferase (β1,6GnT) is shown. The functional features indicated are the disulfide bridges, the glycosylation sites (diamond) and the membrane anchor domain (red box). (B) ClustalW based multiple sequence alignment (MSA) of β1,6GnT with representative proteins from the UniRef90 cluster UniRef90_Q7YQE1. The β1,6GnT sequences of the 2 viruses (from bovine herpesvirus type 4 (BHV-4, marked by arrows) are shown. All functional features that are shown in (A) are fully conserved. (PPT) [file pcbi.1002364.s001.ppt]

## Slide 1
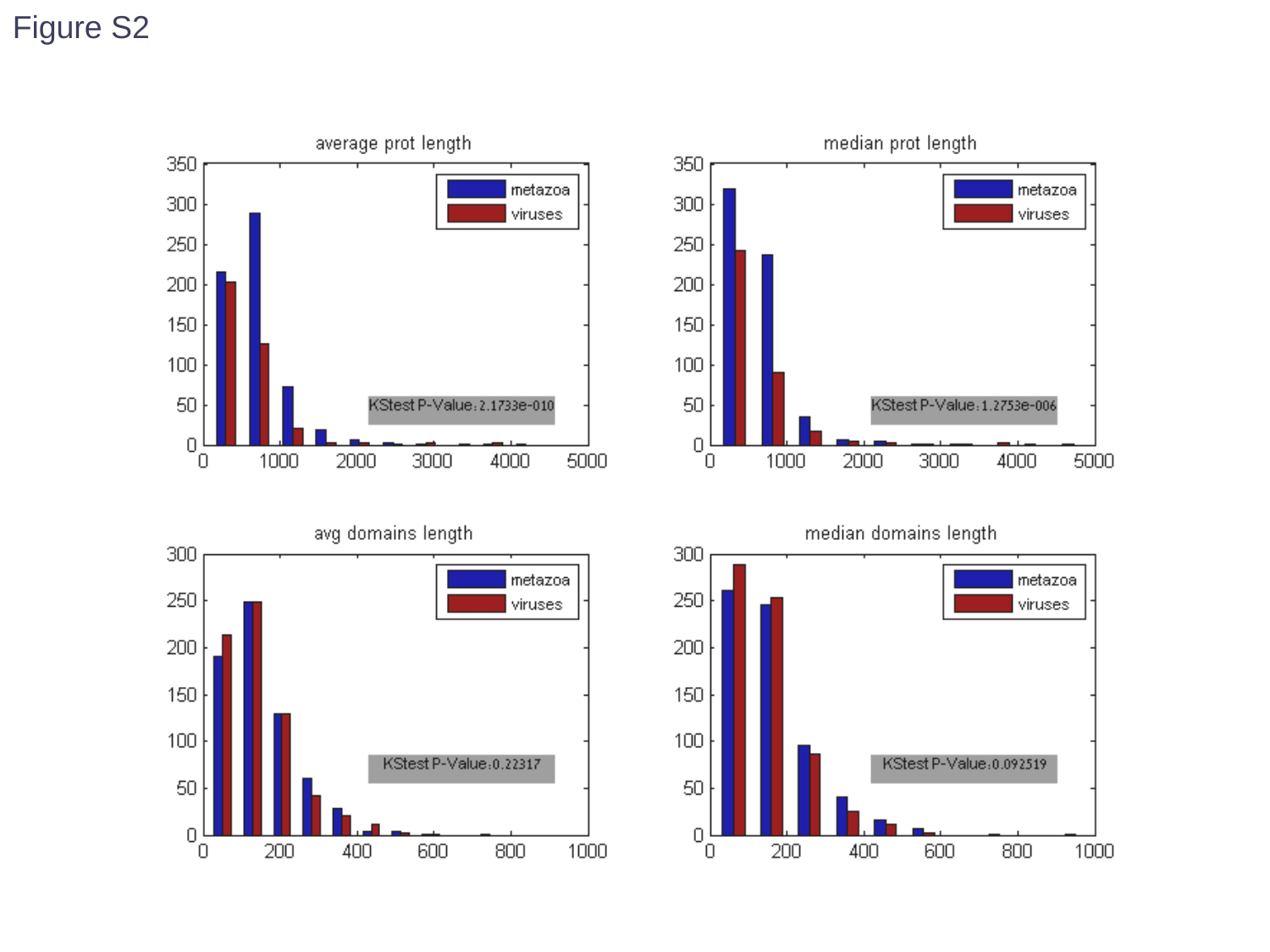

Figure S2

Supplement: Figure S2 — Statistical analysis of protein lengths and Pfam domains. Analysis was performed for a collection of 667 analyzed cross-taxa Pfam entries (Supportive data Table S3). The graphs show the distribution of averages proteins length (two distributions per each Pfam family: one for the metazoan proteins and one for the viral proteins. A statistical KS test was performed on the domains length. No significant difference between the metazoan domains and the counterpart viral domains is detected. The same results were observed when using other statistical tests (e.g., t-test, not shown). The average and median proteins length and the average and median domain length is shown, next to the results of the statistical significant tests. (PPT) [file pcbi.1002364.s002.ppt]

## Slide 1
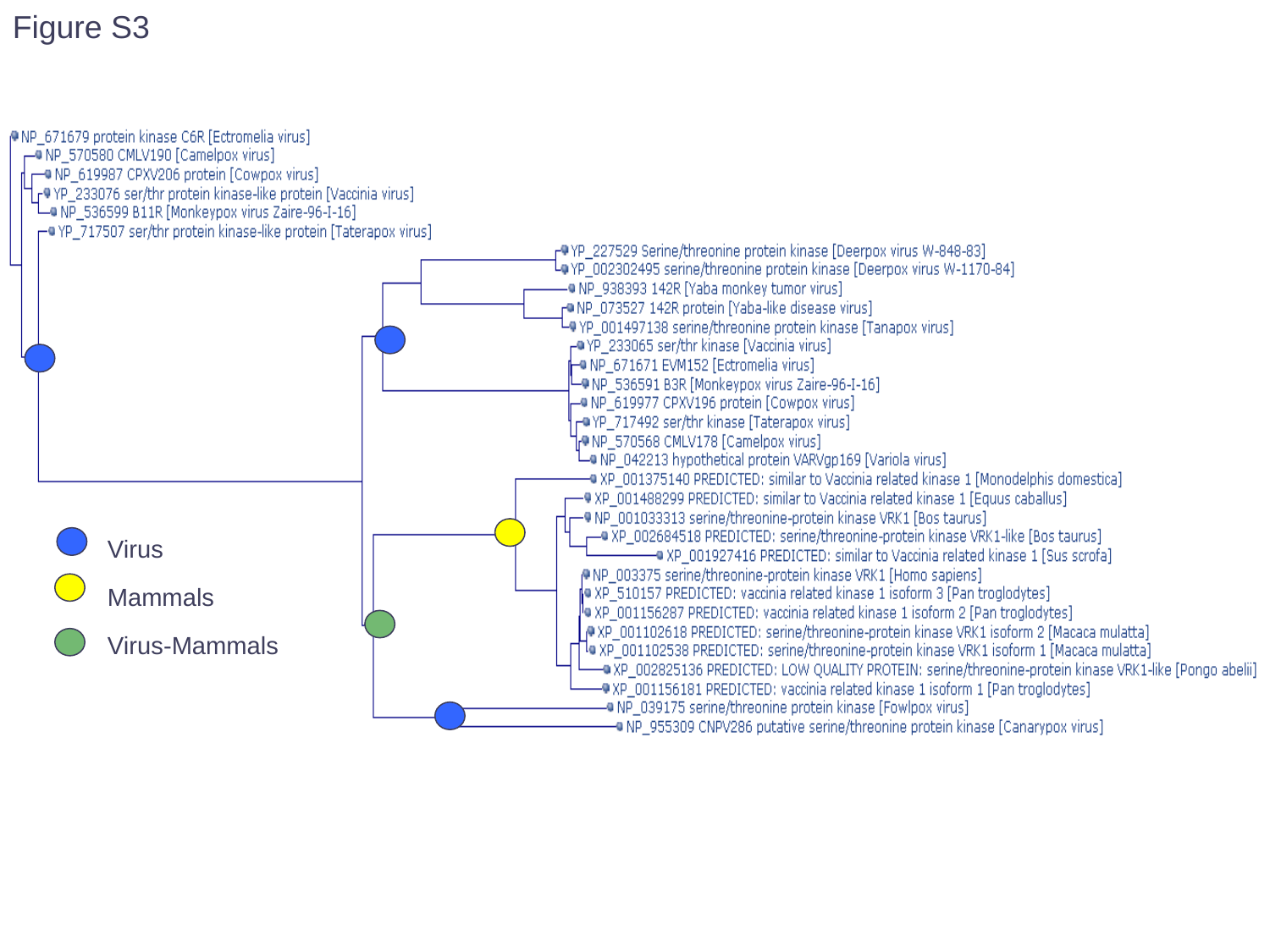

Figure S3
Virus
Mammals
Virus-Mammals

Supplement: Figure S3 — Phylogenetic tree of the viral B1R kinase family. A BLAST search (http://blast.ncbi.nlm.nih.gov) for the 32 highest scored proteins that belong to the B1R kinase family is shown. The query protein used is protein kinase CMLV190 from Camelpox virus. All viruses that were identified belong to dsDNA Class I of different genera. The tree branches are color coded for viruses and mammals (including platypus). All the 21 viral sequences belong to dsDNA Class I from different genera. Representatives are of Orthopoxvirus (Variola, cowpox virus) Capripoxvirus (e.g., Lumpy skin disease virus), Leporipoxvirus (Rabbit fibroma virus) and Yatapoxvirus (e.g., Yaba monkey tumor virus) and more. (PPT) [file pcbi.1002364.s003.ppt]
