# Supplementary material for: Viral Proteins Acquired from a Host Converge to Simplified Domain Architectures
Source: PLoS Comput Biol. 2012 Feb 2;8(2):e1002364. doi: 10.1371/journal.pcbi.1002364 (PMC3271019; doi:10.1371/journal.pcbi.1002364)
Supplement: Figure S4 — Linker lengths in Pfam families that contain viral and metazoan proteins. The cumulative fraction function for all analyzed Pfam families for TAIL and IDOL sequences. A zoomed section of this graph is shown in Figure 6. Viral proteins are marked in red and metazoan proteins in blue. (PPTX) [file pcbi.1002364.s004.pptx]

## Slide 1
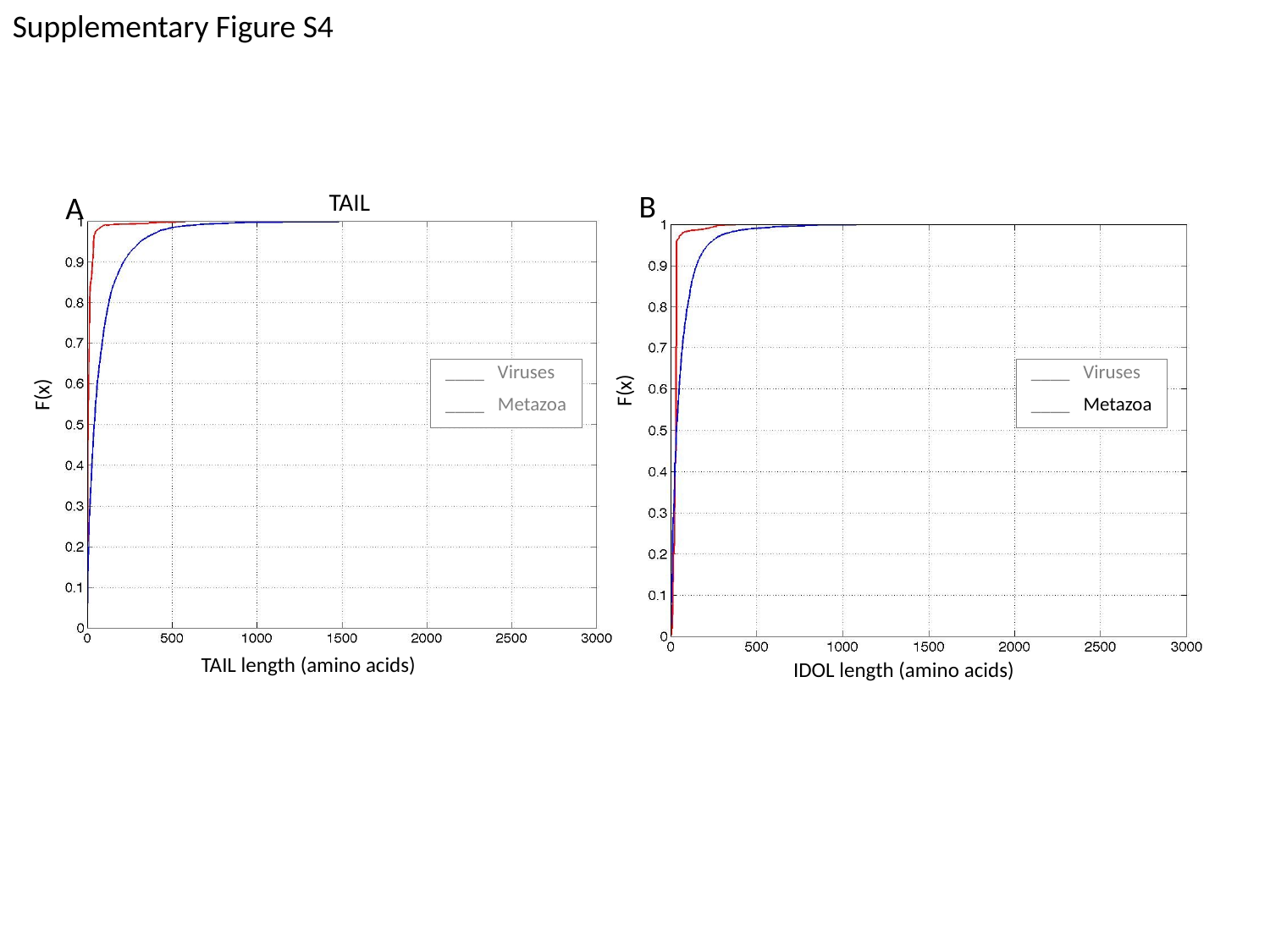

Supplementary Figure S4
B
TAIL
A
IDOL
____ Viruses
____ Metazoa
____ Viruses
____ Metazoa
F(x)
F(x)
TAIL length (amino acids)
IDOL length (amino acids)
